# Risk of gastric adenoma and adenocarcinoma in patients with familial adenomatous polyposis in Japan: a nationwide multicenter study

**DOI:** 10.1007/s00535-023-02074-8

**Published:** 2024-01-23

**Authors:** Kazuhito Sasaki, Kazushige Kawai, Hiroaki Nozawa, Soichiro Ishihara, Hideyuki Ishida, Keiichiro Ishibashi, Yoshiko Mori, Satoki Shichijo, Yasuhiro Tani, Yoji Takeuchi, Akiko Chino, Misato Takao, Kenji Fujiyoshi, Takaaki Matsubara, Yasuyuki Miyakura, Fumitaka Taniguchi, Tatsuro Yamaguchi, Kohji Tanakaya, Naohiro Tomita, Yoichi Ajioka

**Affiliations:** 1 Department of Surgical Oncology, Faculty of Medicine, The University of Tokyo, 7-3-1 Hongo, Bunkyo-Ku, Tokyo, 113-8655 Japan; 2 The Committee of Hereditary Colorectal Cancer, Japanese Society for Cancer of the Colon and Rectum, Tokyo, Japan; 3 Department of Surgery, Tokyo Metropolitan Cancer and Infectious Diseases Center Komagome Hospital, Tokyo, Japan; 4 Department of Digestive Tract and General Surgery, Saitama Medical Center, Saitama Medical University, Saitama, Japan; 5 Department of Gastrointestinal Oncology, Osaka International Cancer Institute, Osaka, Japan; 6 Department of Genetic Oncology, Division of Hereditary Tumors and Department of Gastrointestinal Oncology, Osaka International Cancer Institute, Osaka, Japan; 7 Department of Gastroenterology, Cancer Institute Hospital of Japanese Foundation for Cancer Research, Tokyo, Japan; 8 Department of Surgery, Kurume University School of Medicine, Kurume, Japan; 9 Department of Surgery, Hyogo College of Medicine, Hyogo, Japan; 10 Department of Surgery, Saitama Medical Center Jichi Medical University, Saitama, Japan; 11 Department of Surgery, National Hospital Organization Iwakuni Clinical Center, Yamaguchi, Japan; 12 Department of Clinical Genetics, Tokyo Metropolitan Cancer and Infectious Diseases Center Komagome Hospital, Tokyo, Japan; 13 Department of Surgery, Toyonaka Municipal Hospital, Toyonaka, Japan; 14 Division of Molecular and Diagnostic Pathology, Niigata University Graduate School of Medical and Dental Sciences, Niigata, Japan

**Keywords:** Familial adenomatous polyposis, Gastric adenoma, Gastric cancer

## Abstract

**Background:**

Patients with familial adenomatous polyposis (FAP) have an increased risk of developing gastric neoplasms. However, the clinical course of FAP with these gastric lesions has not yet been fully clarified. The present study aimed to clarify the changes in the incidence risk of developing gastric adenoma or gastric cancer during the lifespan of patients with FAP.

**Methods:**

Four hundred forty-three patients with data regarding gastric adenoma and gastric cancer retrospectively registered in a nationwide Japanese multicenter study were enrolled. The cumulative incidences and hazard rates (HRs) of gastric neoplasms were evaluated.

**Results:**

The cumulative incidence rates in 50-year-old patients with FAP were 22.8% for gastric adenoma and 7.6% for gastric cancer, respectively. No significant association was found between gastric neoplasms and the colonic phenotype. The peak age for the HR of gastric adenoma was 65 years, with the highest HR (0.043). Regarding the incidence of gastric cancer, the HR increased moderately up to the age of 40 years, but the increase accelerated from the age of 50 years (HR = 0.0067).

**Conclusion:**

Careful surveillance of the upper gastrointestinal tract in elderly patients with FAP, such as shortening the interval of follow-up according to age, may be helpful for early diagnosis of gastric cancer.

**Supplementary Information:**

The online version contains supplementary material available at 10.1007/s00535-023-02074-8.

## Introduction

Familial adenomatous polyposis (FAP) is an autosomal dominant disease caused by a pathogenic variant in the *APC* [1–3]. FAP is the second most common predisposition to hereditary colorectal cancer (CRC) and is characterized by the development of multiple colorectal adenomas in the colon and rectum. Large epidemiological polyposis registries have documented FAP prevalence between 1:20,000 and 1:10,000 in Western countries and 1:17,400 in Japan [4, 5]. The natural history of FAP is characterized by the development of adenomatous polyps in one’s teens [6]. If FAP is left untreated, it is most likely to develop into CRC [7]. The *APC* germline variant is reportedly undetected in 20–40% of patients with FAP [8, 9].

Upper gastrointestinal lesions, including fundic gland polyposis (FGP), gastric adenoma, gastric cancer, and duodenal adenoma, develop in patients with FAP. Although gastric cancer is not cited as a health risk in Western patients with FAP with a reported lifetime risk of 0.6%, similar to the general population risk [10], the relative risk of gastric cancer increased 2.4 times for males and 4.7 times for females compared with the general population in Japanese patients with FAP [11]. Therefore, surveillance using upper gastrointestinal endoscopy is recommended for the early detection of malignant diseases, such as gastric cancer, in patients with FAP [12].

Previous studies have reported a high incidence of gastric adenoma and gastric cancer in Asian patients with FAP [13, 14]. Although the Kaplan–Meier method used for conventional analysis can visualize cumulative incidences of gastric adenoma or gastric cancer, it is not suitable for analyzing the changes in risk overtime. Alternatively, a hazard function analysis that plots the time change on the horizontal axis and the risk rate on the vertical axis can show the time-dependent aspect of the incidence risk [15, 16]. Therefore, in this study, we aimed to investigate the changes in the incidence risk for developing gastric adenoma or gastric cancer during the lifespan of patients with FAP using hazard function analysis.

## Methods

### Ethics statements

The ethical committees of the Japanese Society for Cancer of the Colon and Rectum (JSCCR) (90–5) and the University of Tokyo (2019112NI) approved this study. This study was performed in accordance with the provisions of the Declaration of Helsinki.

### Study design and patients

This study included Japanese patients with FAP who were listed in the database of the working group of the JSCCR’s “multicenter retrospective study of FAP patients”. Data were retrospectively collected from 35 JSCCR member institutions that are leading hospitals for colorectal treatment in Japan. The included patients were diagnosed with FAP before 2018. The diagnostic criteria for FAP included a clinical diagnosis of FAP. The criteria for the clinical diagnosis of FAP were as follows: (1) approximately ≥ 100 adenomas in the colorectum, regardless of family history, and (2) < 100 adenomas; however, the patient had a family history of FAP. In addition, patients with *APC* variants classified as likely pathogenic or pathogenic were included. We defined patients with ≥ 100 adenomas in the colon and rectum as having classical FAP, and those with < 100 adenomas as having attenuated FAP (AFAP).

The criteria for surgery, follow-up methods, and surveillance for patients with FAP were determined by each institution and physician.

### Statistical analysis

Cumulative cancer risks were calculated using the Kaplan–Meier method, and the log-rank test was used to compare the risk of gastric adenoma or gastric cancer. Hazard function analysis for the incidence of gastric adenoma or gastric cancer was performed using R version 3.6.1 with the muhaz package (R Project for Statistical Computing, Vienna, Austria; http://www.r-project.org/) [15, 16]. The hazard function describes the instantaneous incidence rate for gastric adenoma or gastric cancer if the individual survives until a certain time. The time scale was discretized by 1 year. Kaplan–Meier survival estimates and statistical analysis were performed using JMP PRO version 14.2.0 software (SAS Institute Inc., Cary, NC, USA), and statistical significance was defined at *p* < 0.05.

## Results

The database included 505 patients with FAP. Patients with missing data from upper gastroenteroscopy (*n* = 61) or the follow-up (*n* = 1) were excluded. Thus, the total study population comprised 443 patients (Supplemental data 1). The characteristics of the 443 patients with FAP enrolled in this study are presented in Table 1. The median follow-up period from FAP diagnosis was 12.3 years. A total of 300 and 62 patients had classical FAP and AFAP, respectively. Genetic analysis of the *APC* was performed in 221 patients, and germline pathogenic or likely pathogenic variants in the *APC* were detected in 165 patients. In total, 270 patients underwent proctocolectomy or total colectomy for the treatment of FAP, and CRC was diagnosed in 197 patients. Gastric adenoma and gastric cancer were diagnosed in 107 and 58 patients, respectively. Concerning the *Helicobacter pylori* (*H. pylori*) infection, 21 patients had a positive result, 208 had a negative result, and 214 had no data.

**Table 1:** Clinical Characteristics of the 443 patients with familial adenomatous polyposis

Factors		*n* (%)
Sex	Male	214 (48.3)
	Female	229 (51.7)
Age at diagnosis, median		27 (0–78)
Polyp density	Classical (≥ 1001 polyps)	58 (13.1)
	(100–1000 polyps)	242 (54.6)
	Attenuated (≤ 99 polyps)	62 (14.0)
	Data missing	81 (18.3)
Median follow-up duration: years (range)		12.3 (0.3–83.5)
Genetic testing	Yes	221 (49.9)
*APC* variants	Yes	165 (74.7)
Diagnosis of colorectal cancer	Yes	192 (43.3)
Number of patients who underwent proctocolectomy or total colectomy		270 (60.9)
*Helicobacter pylori* infection	Positive	21 (4.7)
	Negative	208 (47.0)
	Data missing	214 (48.3)
Gastric adenoma	Yes	107 (24.3)
Gastric cancer	Yes	58 (13.1)

The frequencies of FGP, gastric adenoma, gastric cancer, duodenal adenoma, thyroid cancer, desmoid tumor, and jaw osteoma were 65.4%, 24.3%, 13.1%, 54.0%, 4.5%, 13.6%, and 4.5%, respectively (Table 2).

**Table 2:** Extracolonic diseases in the 443 patients with familial adenomatous polyposis

Type of diseases	Number of patients (%)
Fundic gastric polyposis	289 (65.4)
Gastric adenoma	107 (24.3)
Gastric cancer	58 (13.1)
Duodenal adenoma	239 (54.0)
Thyroid cancer	20 (4.5)
Desmoid tumor	60 (13.6)
Jaw osteoma	20 (4.5)

**Fig. 1:**
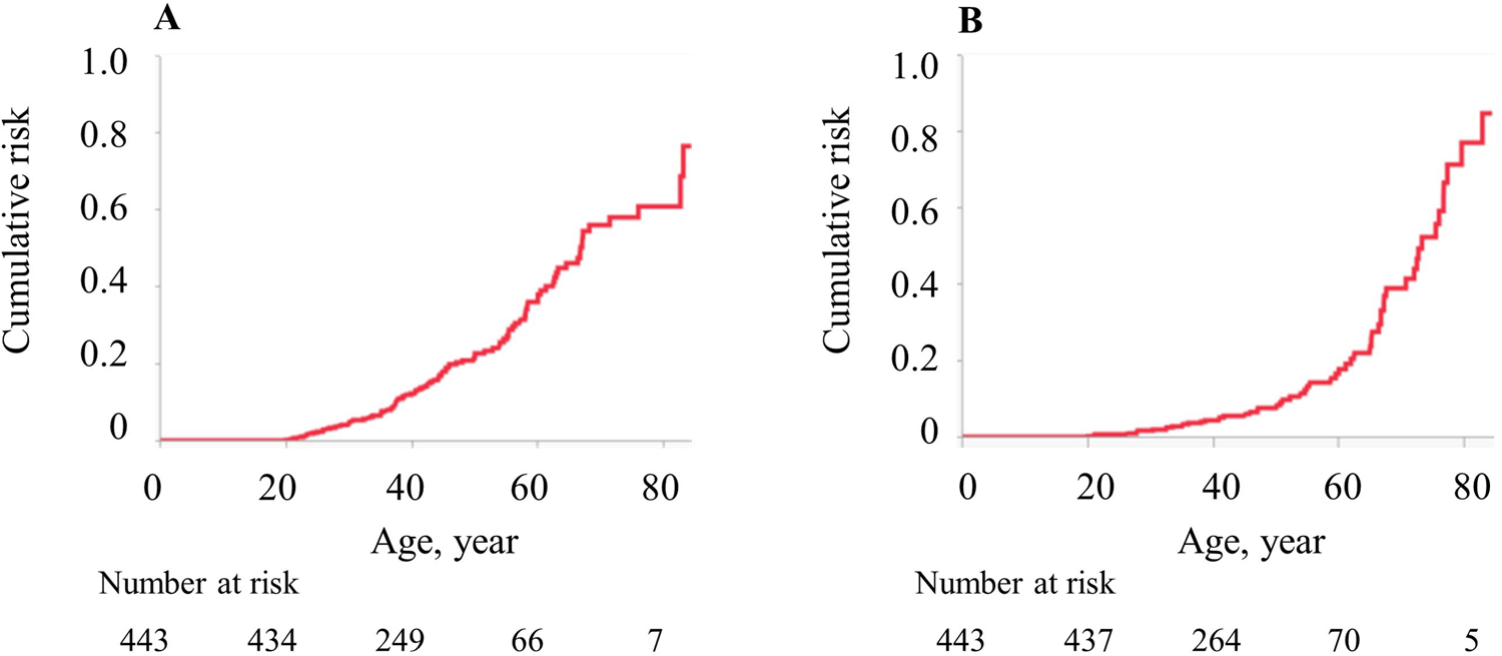
Cumulative incidence of upper gastrointestinal polyps and tumors in 443 patients with familial adenomatous polyposis. **A** Gastric adenoma, **B** gastric cancer

**Fig. 2:**
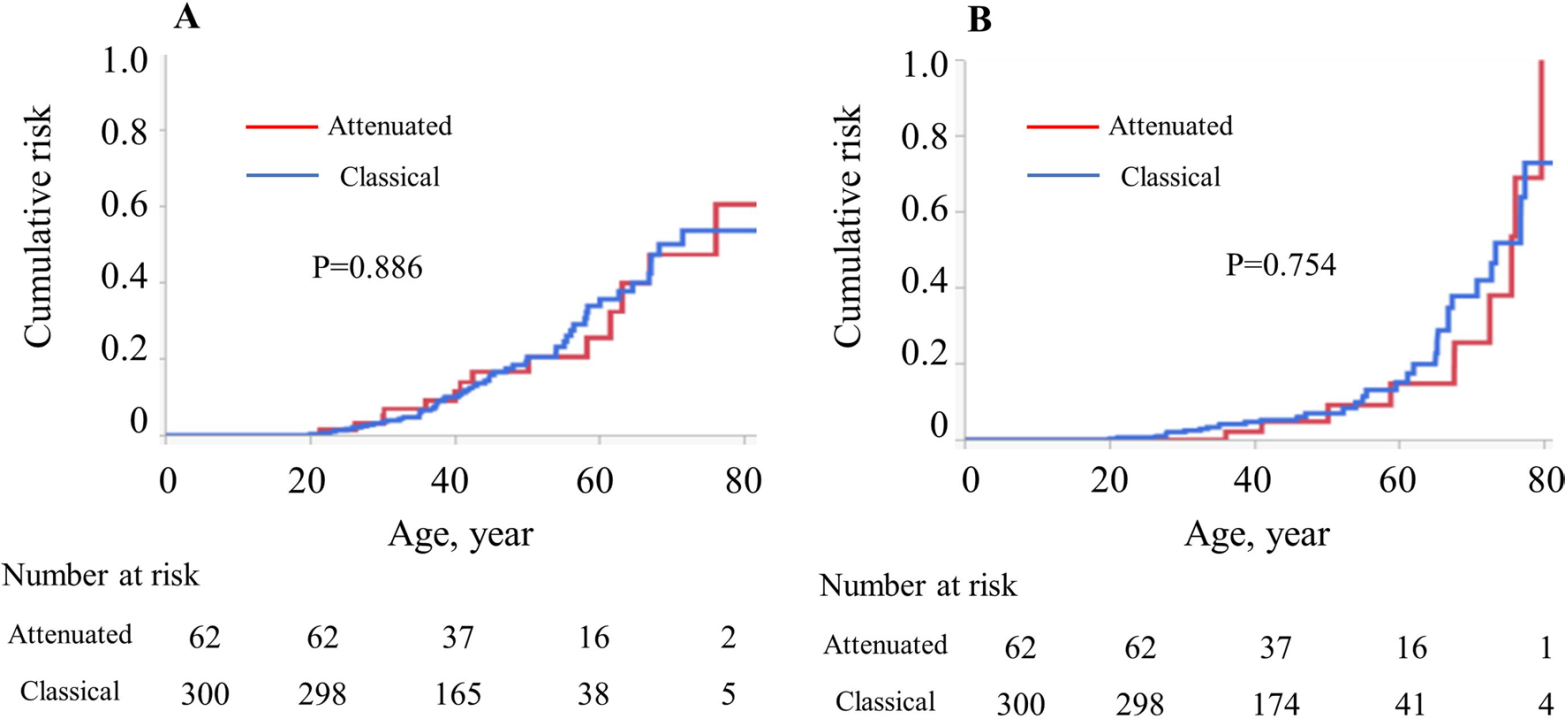
Cumulative incidence of upper gastrointestinal polyps and tumors in 362 patients with familial adenomatous polyposis by colonic phenotype. **A** Gastric adenoma, **B** gastric cancer

**Fig. 3:**
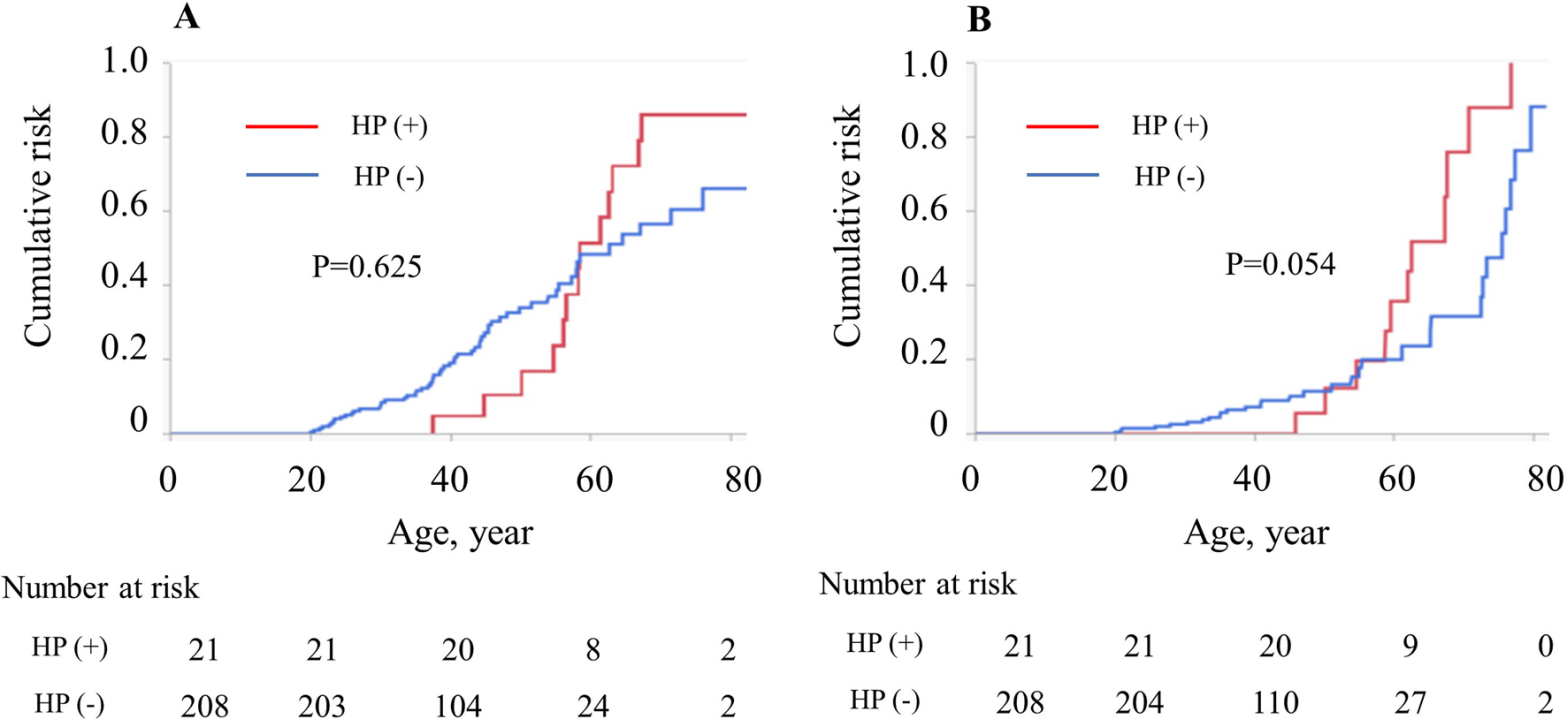
Cumulative incidence of upper gastrointestinal polyps and tumors in 237 patients with familial adenomatous polyposis by the presence/absence of *Helicobacter pylori* infection. **A** Gastric adenoma, **B** gastric cancer

The cumulative incidence rates of gastric adenoma and gastric cancer in 50-year-old patients with FAP were 22.8% and 7.6%, respectively (Fig. 1A, B). When comparing the colonic phenotype between classical FAP and AFAP, the cumulative incidence rates of gastric adenoma in 60-year-old FAP patients were 33.8% for the classical type and 25.7% for AFAP (*p* = 0.886), and the rates of gastric cancer were 15.2% and 14.9% (*p* = 0.754), respectively (Fig. 2A, B). Interestingly, Fig. 2 shows that the cumulative incidence curves between the classical and attenuated types were similar for both gastric adenoma and gastric cancer. Data concerning *H. pylori* infection were missing in 219 patients. In the other patients, the cumulative incidence rates of gastric adenoma in 60-year-old FAP patients were 51.6% in *H. pylori*-positive and 48.3% in *H. pylori*-negative (*p* = 0.625), respectively. The rates of gastric cancer were 35.7% in *H. pylori*-positive and 19.9% in *H. pylori*-negative (*p* = 0.054), respectively (Fig. 3).

**Fig. 4:**
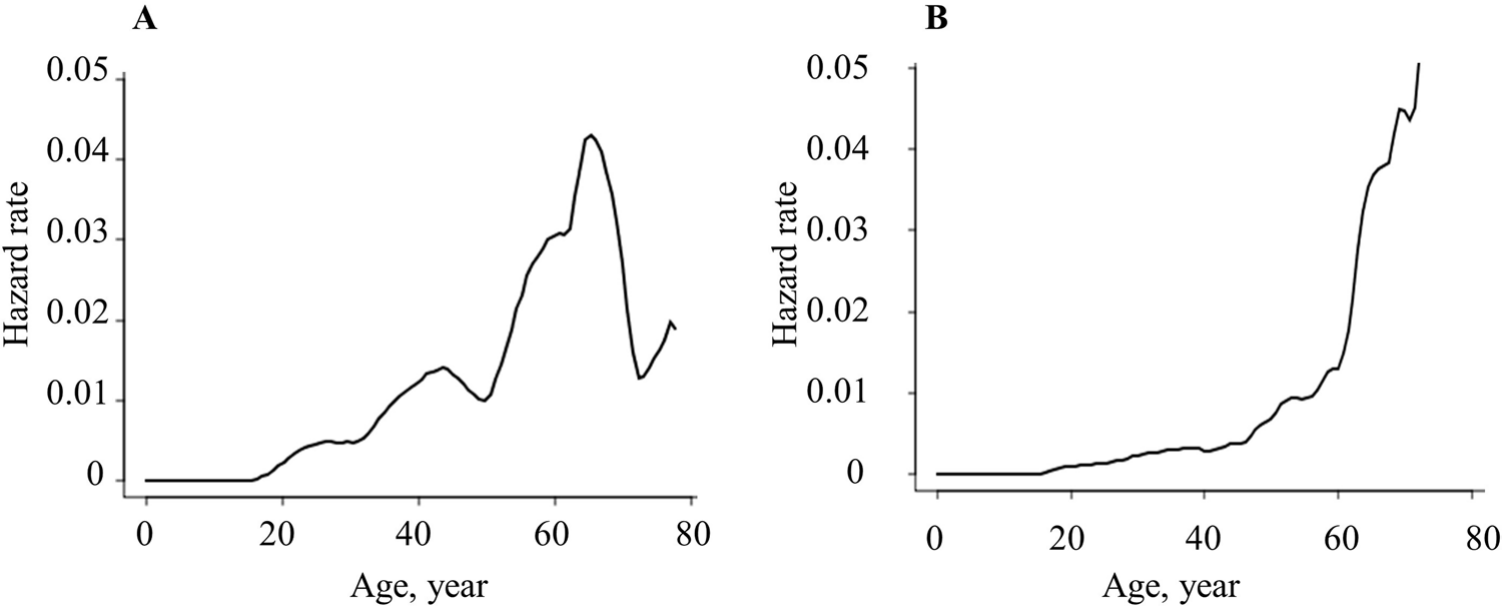
Hazard function plots for developing **A** gastric adenoma and **B** gastric cancer

Figure 4A, B shows a hazard function plot of the incidence of gastric adenoma and adenocarcinoma. Table 3 shows the HR according to age. The peak age for the HR of gastric adenoma was 65 years, with the highest HR of 0.043, and the HR decreased thereafter. Regarding the incidence of gastric cancer, the HR increased moderately up to the age of 40 years, but the increase accelerated from ≥ 50 years.

**Table 3:** Results of hazard function analysis

Age (years)	Gastric adenoma hazard rate	Gastric cancer hazard rate
10	0.0000	0.0000
20	0.0019	0.0010
30	0.0048	0.0023
40	0.0120	0.0029
50	0.0100	0.0067
60	0.0304	0.0131
70	0.0271	0.0447

## Discussion

Extracolonic diseases such as gastric cancer, duodenal cancer, and desmoid tumor, are a major cause of fatality in patients with FAP in Japan [12, 17]. Considering the high risk for gastric cancer, careful monitoring with long-term follow-up is necessary. This is the first study to use hazard function analysis to determine the time course of the HR for the incidence of gastric cancer and gastric adenoma using the data of patients with FAP from a multicenter database. Our findings highlight that the increase in HR for gastric cancer was accelerating for those aged ≥ 50 years.

In this study, the cumulative incidence rate and HR of gastric cancer in 50-year-old patients were 7.6% and 0.0067, respectively. In the general population of Japan, gastric cancer accounted for almost half of all cancer-related deaths in the 1960s, but this proportion continues to decline. According to the 2019 cancer statistics forecast by the National Cancer Center Cancer Information Service [18], gastric cancer ranks third in the number of deaths, after lung and colorectal cancers. The incidence rates of gastric cancer at approximately 50 years of age are 0.00037 in men and 0.00019 in women in the general population. Additionally, the incidence rate increased moderately up to the age of 50 years; however, this increase accelerated from 50 to 80 years. Interestingly, the age-related increase in the gastric cancer incidence was similar between patients with FAP and the general population. Furthermore, comparing the HR of 0.0067 in patients with FAP and the incidence rate of 0.00037 in men in the general population at the age of 50 years, the risk for gastric cancer is much higher in patients with FAP than that in the general population. Therefore, careful surveillance of patients with FAP is important for the detection and treatment of gastric cancer.

Whether the surveillance program for gastric cancer should be the same for classical FAP or AFAP is unclear. Some reports have been published regarding the correlation between the upper gastrointestinal phenotype and *APC* genotype. Miyaki et al. showed that gastric adenoma and duodenal adenoma development are related to variants in codons 1450–1564 of the *APC* [19]. Since *APC* germline variants in this region are associated with the classical colonic phenotype [19], gastric and duodenal adenomas are more frequently found in patients with classical FAP than in those with AFAP. In contrast, Sample et al. showed that the number of gastric polyps, including gastric adenoma and fundic gastric polyps, did not differ based on the genotype; however, advancing age was correlated with the severity of gastric polyposis [20]. The analysis included cases of severe and mild upper gastrointestinal phenotypes in patients with identical *APC* variants. It was suggested that the location of the *APC* variant is not absolutely predictive of an upper gastrointestinal phenotype [20]. Similarly, our study showed that the incidence rates of gastric adenoma and adenocarcinoma were similar between classical FAP and AFAP. In addition, they were strongly correlated with advanced age. It is well known that *H. pylori* is an important etiologic agent for gastric cancer in the general population [21]. Although our study showed that the development of gastric cancer tended to be related to *H. pylori* infection in Japanese FAP patients, the data were not available in half of the patients. Moreover, the definitions and criteria for *H. pylori* infection were not the same for all patients due to historical background. According to our data, the *H. pylori* infection rate was 4.7%, which is very low compared to the general infection rate among Japanese people (40%) [22]. Due to this lack of data, we could not accurately show the association between *H. pylori* infection and the occurrence of gastric cancer in patients with FAP. Further studies are needed to elucidate the complex relationship between the incidence of gastric tumors in patients with FAP and the genotype, the colonic phenotype, and clinical characteristics including age, sex, and *H. pylori* infection.

Gastric cancer screening is a substantial problem for patients with FAP. To date, no prospective studies have investigated the efficacy of upper gastrointestinal tract surveillance in patients with FAP. The Japanese guidelines strongly recommend upper gastrointestinal surveillance in patients with FAP because of the increased risk of gastric and duodenal cancers compared to that in the general population [12]. Baseline gastroscopy is initiated at 20–25 years of age and is repeated regularly once a year [12]. In the general population of Japan, gastroscopy is recommended as a population-based screening method according to the revised 2014 Japanese Guidelines for Gastric Cancer Screening [23], and this recommendation has changed from once a year for individuals aged > 40 years to once every 2 years for individuals aged > 50 years, reflecting the recent decline in gastric cancer mortality by age group. Our study showed that the development of gastric cancer was strongly accelerated in patients aged > 50 years, similar to the general population. Furthermore, the incidence of gastric cancer in patients with FAP is much higher than that in the general population. Therefore, we suggest that gastroscopy for gastric cancer screening should be recommended once a year in middle-aged individuals and shortening the interval of follow-up with age.

Regarding gastric surveillance for patients with FAP, it is important to know the endoscopic features of gastric neoplasms in these patients. Most gastric cancers in patients with FAP occur in an area of polyposis in the proximal gastric body or fundus [24]. Kunnathu et al. reported that the presence of a white mucosal patch in the stomach of a FAP patient is a feature associated with gastric cancer because 2/14 (14.3%) patients had proximal gastric cancer [25]. Among the histologic phenotypes, high-grade dysplasia was present in 41% of patients with FAP with a whitish area of the proximal stomach [26]. Interestingly enough, Nakano K et al. reported the relationship between the atrophic gastritis status (AG) and gastric neoplasms in patients with FAP [27]. They showed that gastric neoplasms in the AG-negative group were frequently located in the upper-third of the stomach, and all neoplasms in the AG-negative group were whitish whereas most neoplasms (65%) in the AG-positive group were reddish. In addition, all gastric neoplasms in AG-negative group showed the gastric phenotype on immunophenotype analysis. In sporadic cases, the gastric phenotype had a high potential for malignancy [28]. Therefore, in patients with FAP, early detection and treatment of gastric neoplasms which were whitish areas, in the AG-negative group may have clinical implication to improve prognosis. Considering these endoscopic features, we assume that intensive surveillance by an expert was recommended for FAP patients with these endoscopic features.

Concerning gastric adenoma in patients with FAP, our results showed that the HR of gastric adenoma decreased in patients older than 65 years. In a report on 104 FAP patients with gastric adenoma, many of the patients were relatively young, with a median age of 47 years [29]. Although the incidence of gastric adenoma in the general Japanese population was shown to increase with age [30], based on our results and the previous report [29], gastric adenoma tends to occur earlier in patients with FAP than in the general population. This also suggests that older FAP patients without gastric adenoma may not need intensive gastric surveillance. However, because our data did not include many older patients with FAP, these hypotheses need further verification.

The present study has several limitations. First, it was a retrospective study. However, the validity of the database of the working group of the nationwide Japanese multicenter study has been established [31]. Second, data regarding the details of endoscopic follow-up and morphological features were lacking. The surveillance programs for the upper gastrointestinal tract were not the same. These factors affected the progression of these polyps. Third, the present study included Japanese patients with FAP. Therefore, it is not clear whether our results apply to Western patients with FAP. Another limitation is that the *APC* pathogenic variant and *H. pylori* infection data were unavailable for approximately half of the patients. These data may help to elucidate the development of gastric adenoma and carcinoma in patients with FAP.

This is the first study to report the time course of the HR of the incidence of gastric cancer and gastric adenoma in patients with FAP. Furthermore, we demonstrated an age-related increase in the gastric cancer incidence in patients with FAP, which is higher than that in the general population. Thus, careful surveillance of the upper gastrointestinal tract in elderly patients with FAP, such as shortening the interval of follow-up according to age, may be helpful for early diagnosis of gastric cancer.

### Supplementary Information

Below is the link to the electronic supplementary material.

Supplementary file1 (JPG 354 KB)

## References

[1] Groden J, Thliveris A, Samowitz W (1991). Identification and characterization of the familial adenomatous polyposis coli gene. Cell.

[2] Joslyn G, Carlson M, Thliveris A (1991). Identification of deletion mutations and three new genes at the familial polyposis locus. Cell.

[3] Kinzler KW, Nilbert MC, Su LK (1991). Identification of FAP locus genes from chromosome 5q21. Science.

[4] Bulow S, Faurschou Nielsen T, Bulow C (1996). The incidence rate of familial adenomatous polyposis Results from the Danish Polyposis Register. Int J Colorectal Dis.

[5] Murata M, Utsunomiya J, Iwama T (1981). Frequency of adenomatosis coli in Japan. Jinrui Idengaku Zasshi.

[6] Hyer W, Cohen S, Attard T (2019). Management of Familial Adenomatous Polyposis in Children and Adolescents: Position Paper From the ESPGHAN Polyposis Working Group. J Pediatr Gastroenterol Nutr.

[7] Arvanitis ML, Jagelman DG, Fazio VW (1990). Mortality in patients with familial adenomatous polyposis. Dis Colon Rectum.

[8] Miyoshi Y, Ando H, Nagase H (1992). Germ-line mutations of the APC gene in 53 familial adenomatous polyposis patients. Proc Natl Acad Sci U S A.

[9] Grover S, Kastrinos F, Steyerberg EW (2012). Prevalence and phenotypes of APC and MUTYH mutations in patients with multiple colorectal adenomas. JAMA.

[10] Jagelman DG, DeCosse JJ, Bussey HJ (1988). Upper gastrointestinal cancer in familial adenomatous polyposis. Lancet.

[11] Iwama T, Mishima Y, Utsunomiya J (1993). The impact of familial adenomatous polyposis on the tumorigenesis and mortality at the several organs. Its rational treatment Ann Surg.

[12] Tomita N, Ishida H, Tanakaya K (2021). Japanese Society for Cancer of the Colon and Rectum (JSCCR) guidelines 2020 for the Clinical Practice of Hereditary Colorectal Cancer. Int J Clin Oncol.

[13] Park JG, Park KJ, Ahn YO (1992). Risk of gastric cancer among Korean familial adenomatous polyposis patients. Report of three cases. Dis Colon Rectum.

[14] Yamaguchi T, Ishida H, Ueno H (2016). Upper gastrointestinal tumours in Japanese familial adenomatous polyposis patients. Jpn J Clin Oncol.

[15] Ahmed FE, Vos PW, Holbert D (2007). Modeling survival in colon cancer: a methodological review. Mol Cancer.

[16] Kawai K, Nozawa H, Sasaki K (2022). Hazard function analysis for development of second primary colorectal cancer after surgery for primary colorectal cancer. J Gastroenterol Hepatol.

[17] Kumamoto K, Ishida H, Tomita N (2023). Recent Advances and Current Management for Desmoid Tumor Associated with Familial Adenomatous Polyposis. J Anus Rectum Colon.

[18] National Cancer Center. Center for Cancer Control and Information Services. (https://ganjoho.jp/reg_stat/statistics/stat/cancer/5_stomach.html)

[19] Miyaki M, Yamaguchi T, Iijima T (2008). Difference in characteristics of APC mutations between colonic and extracolonic tumors of FAP patients: variations with phenotype. Int J Cancer.

[20] Sample DC, Samadder NJ, Pappas LM (2018). Variables affecting penetrance of gastric and duodenal phenotype in familial adenomatous polyposis patients. BMC Gastroenterol.

[21] Uemura N, Okamoto S, Yamamoto S (2001). Helicobacter pylori infection and the development of gastric cancer. N Engl J Med.

[22] Ito M, Sugiyama A, Mino M (2022). Prevalence of Helicobacter pylori infection in the general population evaluated by a resident-register-based epidemiological study. J Gastroenterol.

[23] Hamashima C, Systematic Review G (2018). Guideline Development Group for Gastric Cancer Screening G. Update version of the Japanese Guidelines for Gastric Cancer Screening. Jpn J Clin Oncol.

[24] Mankaney G, Leone P, Cruise M (2017). Gastric cancer in FAP: a concerning rise in incidence. Fam Cancer.

[25] Kunnathu ND, Mankaney GN, Leone PJ (2018). Worrisome endoscopic feature in the stomach of patients with familial adenomatous polyposis: the proximal white mucosal patch. Gastrointest Endosc.

[26] Shimamoto Y, Ishiguro S, Takeuchi Y (2021). Gastric neoplasms in patients with familial adenomatous polyposis: endoscopic and clinicopathologic features. Gastrointest Endosc.

[27] Nakano K, Kawachi H, Chino A (2020). Phenotypic variations of gastric neoplasms in familial adenomatous polyposis are associated with endoscopic status of atrophic gastritis. Dig Endosc.

[28] Koseki K, Takizawa T, Koike M (2000). Distinction of differentiated type early gastric carcinoma with gastric type mucin expression. Cancer.

[29] Martin I, Roos VH, Anele C (2021). Gastric adenomas and their management in familial adenomatous polyposis. Endoscopy.

[30] Kamiya T, Morishita T, Asakura H (1982). Long-term follow-up study on gastric adenoma and its relation to gastric protruded carcinoma. Cancer.

[31] Matsubara T, Beppu N, Ikeda M (2022). Current clinical practice for familial adenomatous polyposis in Japan: A nationwide multicenter study. Ann Gastroenterol Surg.

