# Supplementary figures and images for: Risk of gastric adenoma and adenocarcinoma in patients with familial adenomatous polyposis in Japan: a nationwide multicenter study
Source: J Gastroenterol. 2024 Jan 23;59(3):187–94. doi: 10.1007/s00535-023-02074-8 (PMC10904405; doi:10.1007/s00535-023-02074-8)

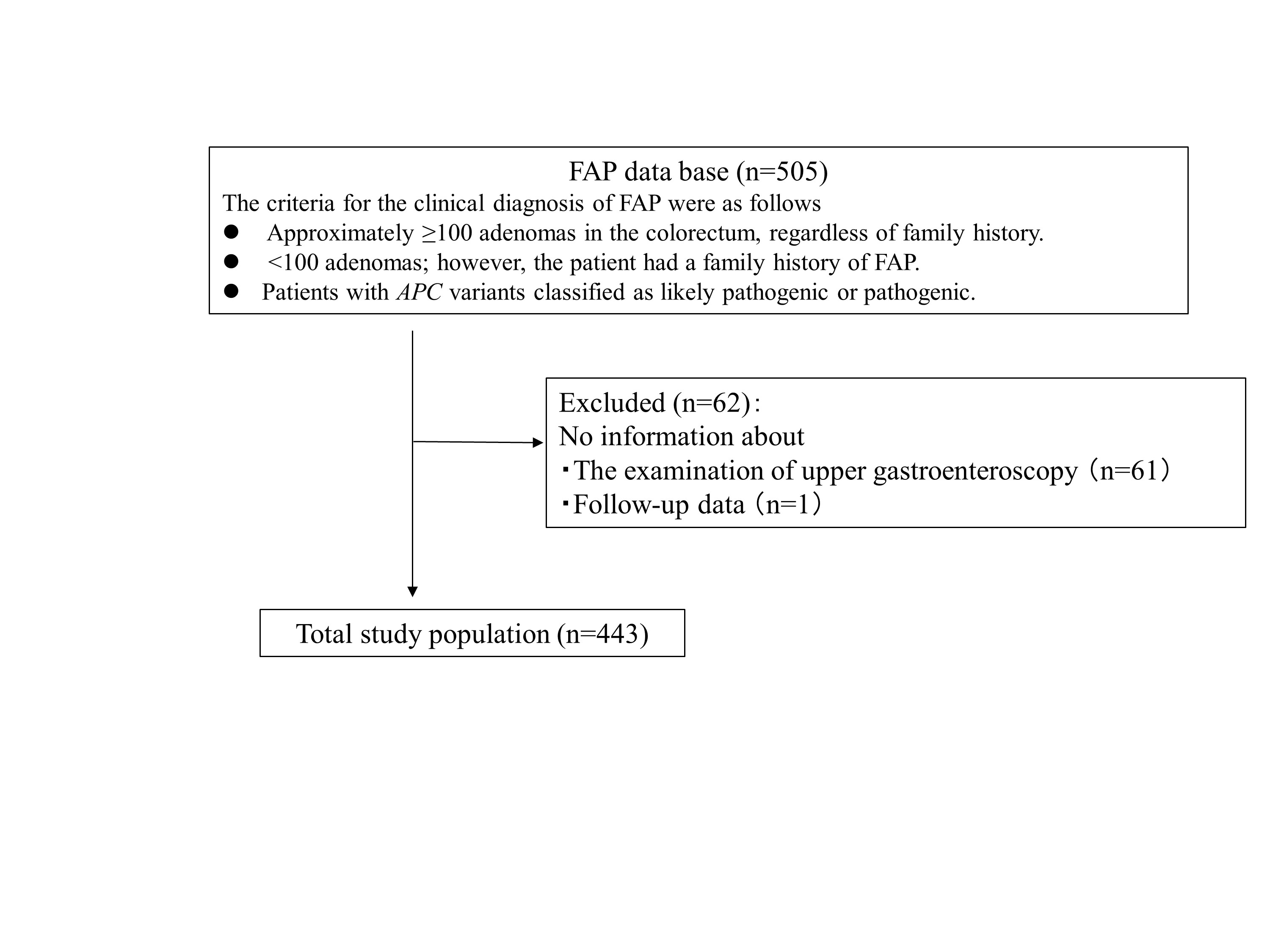

Supplement: Supplementary file 1 — Supplementary file1 (JPG 354 KB) [file 535_2023_2074_MOESM1_ESM.jpg]
